# When social media hurts: a nine-month prospective study on self-blame as a mediator between problematic social media use and suicidal ideation in adolescents

**DOI:** 10.1007/s00787-026-03015-5

**Published:** 2026-03-30

**Authors:** Cirenia Quintana-Orts, Carolina Yudes, Víctor Sánchez-Moreno, Lourdes Rey

**Affiliations:** 1 Department of Developmental and Educational Psychology, Faculty of Psychology and Speech Therapy, University of Malaga, Málaga, Spain; 2 Department of Personality, Assessment, and Psychological Treatment, Faculty of Psychology and Speech Therapy, University of Malaga, Málaga, Spain

**Keywords:** Problematic social media use, Emotion regulation, Suicidal ideation, Adolescence, Mediation

## Abstract

The increasing prevalence of problematic social media use (PSMU) has heightened concerns about its adverse impact on social media users’ mental health, and specifically on suicidality. Despite reviews investigating the associations between PSMU, emotion regulation, and suicidality, there is a lack of understanding of the potential role that specific emotion regulation might play. This study aimed to bridge this gap by examining the mediating role of four specific maladaptive cognitive emotion regulation strategies (CERS) (i.e., rumination, self-blame, other-blame, catastrophizing) in the prospective link between PSMU and suicidal ideation in a sample of 517 adolescents (M = 13.41, SD = 1.06). The results showed that PSMU was significantly associated with suicidal ideation nine months after its assessment. Maladaptive CERS were negatively associated with both PSMU and suicidal ideation, with the exception of other-blame, which was not significantly related to PSMU. The findings of mediation analyses indicated that only self-blame mediates the negative and prospective link between PSMU and suicidal ideation. Specifically, those adolescents who present PSMU were found to exhibit a higher level of self-blame, which, in turn, contributed to greater suicidal ideation nine months later. These findings reinforce the notion that not only might PSMU be a risk factor for suicide in adolescence, but also that efforts should focus on identifying such adolescents and on helping them to reduce self-blame. The implications of this study for the prevention of suicidal ideation associated with PSMU in adolescents are discussed.

## Introduction

Suicide is the third leading cause of nonnatural death among adolescents [1], underscoring the critical need to understand suicidality—a broad term encompassing suicidal ideation, attempts, and behaviors. Suicidal ideation specifically refers to thoughts about death and self-harm, ranging from fleeting considerations to detailed plans for taking one’s own life [2]. Meta-analytical research has estimated that 14–23% of individuals under the age of 22 worldwide have experienced active suicidal ideation at some point in their lives [3]. Most alarming are the findings from longitudinal studies, which have revealed that persistent suicidal ideation, characterized by a high frequency or prolonged duration, is the strongest predictor of future suicidal attempts and behaviors in young people [4], highlighting the need for further research into its underlying mechanisms and risk factors.

This global public health problem arises from a complex interplay of individual, interpersonal, and sociocommunity factors [5], ultimately reflecting an individual’s effort to escape circumstances perceived as overwhelming or hopeless [6]. Unfortunately, most theories on suicidality have focused on adults, overlooking the unique neurodevelopmental challenges of adolescence. This stage involves significant physical, cognitive, psychosocial, and emotional maturation [7], during which the imbalance between developed and maturing brain areas makes adolescents more prone to sensation-seeking, impulsive and risky behaviors, and increased sensitivity to social evaluation [8].

All of this, in turn, increases psychological vulnerability by affecting the decision-making process and coping strategies in response to stressful life changes [9]. In this context, evidence from longitudinal studies has consistently identified feelings of loneliness and depressive symptoms during adolescence with an increased risk of suicide and self-harm behaviors [10]. However, emerging research has emphasized additional risk factors, also related to depression, that may further increase vulnerability to suicide. These include impaired stress–response systems [11], negative social media experiences [12], peer-related issues such as cyberbullying [13], and difficulties in expressing and regulating one’s own emotions [14]. This study aims to deepen the understanding of the relationship between some of these factors and their impact on suicidal ideation during this developmental stage, providing insights relevant for targeted, evidence-based interventions focused on enhancing psychological well-being.

## Problematic social media use and suicidal ideation

Problematic social media use (PSMU) is characterized by difficulties in regulating social media engagement [15]. Unlike frequent use of social media, PSMU involves a lack of control, compulsive patterns of engagement, and negative psychological and/or social consequences [16]; features that have led to conceptualizations of PSMU sharing characteristics with behavioral addictions [17]. However, recent evidence suggests that PSMU may not be fully analogous to an addiction, as not all commonly assumed features are equally associated with psychological maladjustment. Distress-driven and coping-related social media use appears to be more closely linked to negative mental health consequences, including suicidal ideation, than indicators such as prioritizing social media or increased time spent online [18].

An expanding body of literature supports the association between PSMU and these adverse outcomes among young people [19], with PSMU emerging as a significant predictor of suicidality [12, 20]. However, this relationship is complex due to the unique characteristics of the variables involved, and understanding how and why adolescents use social media may provide important insights into the mechanisms underlying these outcomes. Different theoretical models have been proposed to explain them, for example, Caplan’s cognitive-behavioral model [21, 22] suggests maladaptive patterns of social media use may be associated with dysfunctional cognitive and behavioral strategies in response to negative events. According to this model, poor self-regulation —driven by a preference for online social interaction and mood regulation—leads to negative outcomes. Nevertheless, the multidimensional nature of PSMU has prompted more integrative approaches [23]. The Differential Susceptibility to Media Effects Model [24], grounded in Bronfenbrenner’s bio-ecological framework, posits that media exposure leads to within-person changes in physiology, emotion, and behavior. These media effects are shaped by individual predispositions, contextual influences, and sociocultural processes, such that some adolescents may be particularly susceptible to PSMU depending on how these effects interact with personal and contextual factors.

Supporting this, social media can exacerbate negative self-evaluation and social comparison, affecting identity formation, self-esteem, and social interactions [25], all of which have been linked to increased depressive symptoms and suicidal ideation [12]. Longitudinal studies suggest that PSMU may contribute to depression [26], which in turn can act as a proximal outcome leading to suicidal ideation or behaviors; conversely, depression may also lead to PSMU [27], as adolescents often turn to social media for emotional support, reinforcing the idea that vulnerable adolescents may use social media as a compensatory coping mechanism [28, 29]. Indeed, longitudinal research suggests that the relationship between social media use and suicidal ideation is dynamic and fluctuates daily. Positive social and emotional experiences on social media, such as social connectedness or supportive interactions, may serve a protective role, whereas days dominated by negative experiences, including cyberbullying, negative social comparison, or fear of missing out, predict increases in suicidal ideation [12].

Taken together, these findings suggest that social media constitutes a salient emotional context during adolescence. Beyond their association with PSMU [30], emotion regulation difficulties—particularly at the cognitive level—have been identified as key predictors of psychological adjustment problems in adolescents, including depression, anxiety, aggression, addiction, and suicidal ideation [31, 32]. From this perspective, cognitive emotion regulation strategies may represent key mechanisms through which PSMU and related online experiences contribute to adolescents’ mental health outcomes.

### Cognitive emotion regulation strategies, PSMU, and suicidal ideation

Cognitive emotion regulation refers to the conscious process employed to manage emotionally arousing information and modulate its intensity, duration or expression [33]. Garnefski et al. (2001) identified nine cognitive-emotion regulation strategies (CERS), four of which are classified as maladaptive: self-blame, other-blame, rumination, and catastrophizing [34]. These strategies have been consistently related to a range of internalizing and externalizing problems, including heightened depressive symptoms, anxiety [33, 35], substance use [36], and broader psychopathological outcomes [37, 38].

Regarding suicidal ideation, existing research often relies on unidimensional or global CERS scores, which overlook the potentially distinct roles of specific strategies. This gap is significant because, although maladaptive CERS have been shown to impact suicidal ideation through perceived burdensomeness, thwarted belongingness or hopelessness [39] in non-clinical samples, the cognitive content specificity model [40] suggests that different CERS may follow unique pathways to suicidal ideation. For instance, rumination and self-blame are primarily associated with depression, while catastrophizing and other-blame relate to anxiety [33], notably rumination predicts suicidal ideation independently of depression [41].

Evidence examining these pathways in adolescents, however, remains scarce. Furthermore, the literature is constrained by a predominance of cross-sectional designs, limiting causal inferences [29]. A recent systematic review noted that only one in ten studies employed a longitudinal approach [31]. Notably, that longitudinal study found that adaptive—but not maladaptive—CERS mediated the relationship between emotional intelligence and suicidal ideation over four months in adolescents [14]. Consequently, there is a critical need for longitudinal research that examines specific strategies to clarify their roles over time.

Theoretically, PSMU may foster maladaptive CERS by intensifying exposure to evaluative and comparison-based interactions. Adolescents spend most of their time viewing peers’ profiles rather than posting their own [42], a passive form of engagement that can trigger upward social comparisons and may both precede and reflect psychological maladjustment [43, 44]. According to Social Comparison Theory [45], these upward comparisons may lead adolescents to internalize perceived failures as personal flaws (self-blame) or engage in rumination [46], effects that are particularly pronounced among adolescents with lower popularity or less positive social feedback [44]. Simultaneously, negative feedback or online exclusion can provoke external attributions of unfairness (other-blame) or catastrophizing [47]. Drawing on the Interpersonal Theory of Suicide [6], these cognitive appraisals may act as catalysts for suicidal ideation by eroding social connection. Specifically, internalizing strategies like self-blame and rumination can exacerbate perceived burdensomeness, while other-blame or catastrophizing may deepen thwarted belongingness [48, 49]. Thus, maladaptive CERS may function as the cognitive mechanism through which PSMU-related distress is transformed into suicidal ideation.

Taken together, evidence suggests that certain CERS may differentially mediate the association between PSMU and suicidal ideation, but the specific mechanisms and temporal dynamics in adolescence have yet to be fully elucidated. To address these gaps, the current study employs a prospective model over a nine-month period to examine the mediating role of specific maladaptive CERS in the relationship between PSMU and suicidal ideation in adolescents. The study has two primary objectives: (1) to investigate the prospective associations between PSMU, maladaptive CERS, and suicidal ideation; and (2) to determine the extent to which specific maladaptive CERS mediate the relationship between PSMU and suicidal ideation. By elucidating the dynamics between these variables, this approach could provide critical insight into the cognitive-emotional pathways linking PSMU to long-term suicidality, contributing theoretically and informing targeted interventions aimed at the prevention of adolescent suicide.

In line with the main objectives of this study, the following hypotheses were proposed:

#### Hypothesis 1

(H1): PSMU at Time 1 (T1) would be positively correlated with the four maladaptive CERS (self-blame, other-blame, rumination, and catastrophizing) at T1 and with suicidal ideation at Time 2 (T2).

#### Hypothesis 2

(H2): Self-blame, other-blame, rumination, and catastrophizing would mediate the association between PSMU at T1 and suicidal ideation at T2; that is, adolescents with a higher PSMU at T1 are expected to report an increased use of these four maladaptive strategies at T1, which in turn will be associated with greater levels of suicidal ideation at T2. To avoid possibly confounded effects from unobserved time-invariant traits such as personality [50] and sociodemographic variables such as gender and age, these variables were used as covariates in the mediation model.

## Method and materials

### Participants

An original sample of 919 adolescents aged 12–18 years was recruited from five secondary schools in the province of Málaga (Andalusia, Spain). A total of 56.26% of those who participated at T1 also took part in T2. The reduction in sample size was mainly due to students completing their final year of compulsory education and leaving the school (thus being unable to participate at T2), difficulties matching cases across T1 and T2 because of incomplete identification codes, and student absences on the day of T2 assessment. Adolescents who did not provide data at both time points were excluded from the longitudinal analyses. The final sample consisted of 517 students (233 boys, 282 girls, 1 transgender, 1 nonbinary; M = 13.41, SD = 1.06). With regard to academic courses, their distribution by grade in compulsory secondary education (grades seven to ten) in T1 was as follows: 34.2% in seventh grade, 30.8% in eighth grade, 34.8% in ninth grade, and 0.2% in tenth grade.

### Instruments

The Spanish version of the Bergen Social Media Addiction Scale (BSMAS) [51, 52] was used to evaluate PSMU. The BSMAS assesses the symptoms of PSMU over the past year according to the theoretical framework of the model of behavioral addictions (salience, tolerance, modification mood, relapse, withdrawal, and conflict) [17]. Each item is rated on a five-point Likert scale (1 = very rarely; 5 = very often). One sample item is: “In the last 12 months, I have spent a lot of time thinking about the social media platforms or planned use of them.” The total scores range from 6 to 30, with higher scores indicating a high risk of PSMU. In the present sample, the Cronbach’s alpha reliability was acceptable (α = 0.77).

The Spanish version of the Frequency of Suicidal Ideation Inventory (FSII) [53, 54] was used. This is a five-item self-report measure that assesses the frequency of suicidal thoughts over the past 12 months, with the items again being answered on a five-point Likert scale (1 = never; 5 = every day). An example item is: “Over the past 12 months, how often have you believed that your life was not worth living?” In the present sample, the Cronbach’s alpha reliability was excellent (α = 0.92).

The Spanish version of the Cognitive Emotion Regulation Questionnaire for adolescents (CERQ-SA) [34, 55] was used to assess cognitive coping, or specifically what participants think after experiencing stressful life events or situations. The CERQ-SA consists of 36 items distributed across nine subscales, each consisting of four items that are answered on a five-point Likert scale (1 = almost never; 5 = almost always). The scores can be obtained by adding up these four items for each subscale (minimal score of 4 and maximal score of 20), with higher scores indicating the use of specific cognitive strategies. For the present study, only the subscales designed to evaluate the maladaptive cognitive strategies were used: *Self-blame* (thoughts that attribute responsibility for the situation experienced to oneself; example item: “I think that it’s all caused by me”); *Other-blame* (thoughts that attribute responsibility for the situation to external factors or another person; example item: “I think that it’s all caused by others”); *Rumination* (repetitively negative thoughts and emotions associated with the event; example item: “Again and again, I think of how I feel about it”); and *Catastrophizing* (thoughts that explicitly emphasize the severity of what has been experienced; example item: “All the time, I think that is the worst thing that can happen to you”). The Cronbach’s alpha was 0.63 for self-blame, 0.73 for other-blame, 0.63 for rumination, and 0.64 for catastrophizing in the present sample.

The Spanish adaptation of the short form of Goldberg’s bipolar adjectives [56, 57] was used to measure personality. This instrument consists of 25 items in which respondents rate their agreement with opposite adjective pairs on a 9‑point Likert-type scale. The instrument provides scores for Extraversion (α = 0.82), Agreeableness (α = 0.76), Conscientiousness (α = 0.81), Neuroticism (α = 0.74) and Openness (α = 0.80), consistent with the Big Five model [58].

### Procedure

This study was part of a larger research project, and ethical approval for the data collection procedure was obtained from the Research Ethics Committee of the University of Malaga (169–2023-H), in accordance with the Declaration of Helsinki (2013) [59]. In line with the study protocol, after contacting each school, approval from the head of the school and previous family consent were obtained to ensure voluntary participation. The data collection was conducted by members of the research team using a paper and pencil format. At the beginning of the administration of the instruments, students were informed about the anonymity and the confidentiality of the data, given clear instructions on how to complete the questionnaires, and made aware of their right to withdraw from the study at any time. To ensure this, the questionnaires were registered with an alphanumeric code, which was registered by students at both times.

### Data analysis

The statistical analyses were carried out using IBM SPSS Statistics version 23.0 (SPSS Inc., Chicago, IL, USA). To handle missing data, the expectation–maximization (EM) algorithm was applied for imputation, as described by Liang and Bentler (2004) [60]. As preliminary analyses, descriptive statistics were calculated for all study variables, and Pearson bivariate correlations were examined to assess the relationships between the evaluated dimensions. To test the research hypotheses, mediation analysis was performed using the PROCESS macro [61]. Prior to conducting these analyses, key OLS regression assumptions were evaluated, including independence, normality, multicollinearity, and homoskedasticity, with the heteroskedasticity-consistent standard error (HC3) estimators being applied [61]. A parallel multiple mediation model (Model 4) was used to explore the role of rumination, self-blame, other-blame, and catastrophizing as mediators in the relationship between PSMU in T1 and suicidal ideation in T2, and age, gender, and personality were included as covariates. To assess the significance of the effects, 95% bias-corrected confidence intervals were estimated using 5,000 bootstrapped samples, and effects were considered significant if the confidence intervals did not include zero. Effect sizes were interpreted using conventional benchmarks for the coefficient of determination proposed by Cohen [62], and for standardized regression coefficients following the guidelines of Peterson and Brown [63].

## Results

### Descriptive analyses

Means, standard deviations, and Pearson correlations are presented in Table 1, and overall, the correlations followed the expected patterns. The four maladaptive CERS at T1 showed significant positive associations with one another and with PSMU at T1. Furthermore, self-blame, rumination, catastrophizing, and PSMU at T1 were significantly and positively correlated with suicidal ideation at T2, whereas other-blame did not exhibit a significant correlation with suicidal ideation.

**Table 1:** Descriptive statistics and Pearson correlations

Measures	Min-Max	M	SD	2	3	4	5	6
1. PSMU^a^ T1^b^	6–28	12.28	4.85	0.24**	0.24**	0.35**	0.24**	0.27**
2. Self-blame T1	1–5	2.77	0.86		0.52**	0.35**	0.10*	0.26**
3. Rumination T1	1–5	3.15	0.88			0.44**	0.25**	0.18**
4. Catastrophizing T1	1–5	2.74	0.93				0.36**	0.19**
5. Other-blame T1	1–5	2.49	0.90					0.06
6. Suicidal ideation T2^c^	1–5	1.70	0.94					

*N* ranged between 438 and 514. ^a^PSMU = Problematic Social Media Use; ^b^T1 = Time 1; ^c^T2 = Time 2; * *p* <.05; ** *p* <.01

### The mediating model

**Fig. 1:**
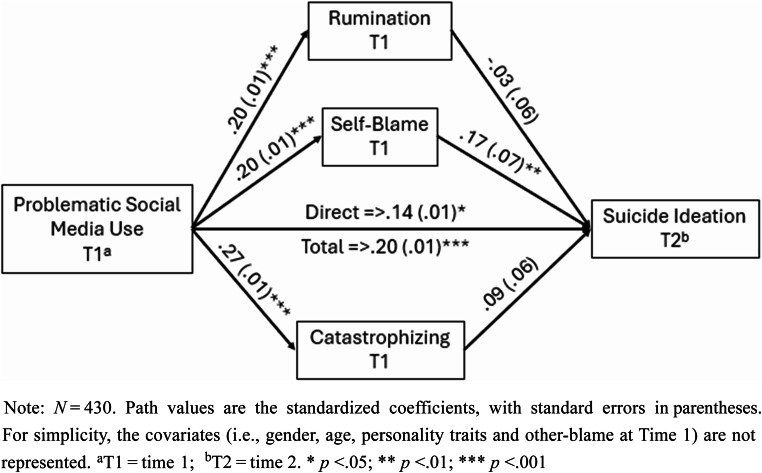
Results of the parallel mediation model

The results of parallel mediation analyses are presented in Table 2; Fig. 1. As other-blame was not significantly correlated with the dependent variable, it was treated as a covariate rather than being included as a mediator. The standardized regression coefficients indicated small‑to‑medium effect sizes for the associations between PSMU at T1 and the three mediators, namely rumination (*β* = 0.20, *p* <.001), self‑blame (*β* = 0.20, *p* <.001) and catastrophizing (*β* = 0.27, *p* <.001), as well as a small effect size for its direct association with suicidal ideation at T2 (*β* = 0.14, *p* <.05). Among the mediators, self-blame was the only variable exhibiting a significant direct effect on suicide ideation (*β* = 0.17, *p* <.01). Furthermore, the indirect effect of PSMU on suicide ideation, mediated through the three maladaptive CERS, was significant only for self-blame (*β* = 0.04, standard error = 0.02, 95% CI: 0.01–0.07). The model accounted for 20% of the variance in suicide ideation at T2 (*R*^*2*^ = 20, *F*(12,417) = 7.11, *p* <.001), indicating a medium overall effect size for the full mediation model.

**Table 2:** Results of parallel mediation analysis

Path/Effect	*β *(SE^a^)	BC 95% CI^b^ (LL-UL)^c^
a-paths (Predictor → Mediators)		
PSMU^d^T1 → Rumination T1^e^	.20 (.01)***	[.02,.06]
PSMU T1 → Self-blame T1	.20 (.01)***	[.02,.06]
PSMU T1 → Catastrophizing T1	.27 (.01)***	[.03,.07]
b-paths (Mediators → Outcome)		
Rumination T1 → Suicidal ideation T2^f^	−.03 (.06)	[−.15,.09]
Self-blame T1 → Suicidal ideation T2	.17 (.07)**	[.06,.32]
Catastrophizing T1 → Suicidal ideation T2	.09 (.06)	[−.02,.21]
Indirect Effects (ab-paths)^g^		
Rumination	−.01 (.01)	[−.03,.02]
Self-blame	.04 (.02)	[.01,.07]
Catastrophizing	.03 (.02)	[−.01,.06]
Total Effect (c-path)^h^	.20 (.01)***	[.02,.06]
Direct Effect (c′-path)^i^	.14 (.01)*	[.01,.05]
Covariates → Suicidal ideation T2		
Gender	.20 (.09)***	[.19,.55]
Age	−.01 (.04)	[−.09,.08]
Other-blame T1	−.01 (.05)	[−.11,.10]
Extraversion	.11 (.04)	[−.02,.14]
Agreeableness	.02 (.04)	[−.07,.09]
Conscientiousness	−.16 (.03)*	[−.15, −.02]
Neuroticism	−.08 (.04)	[−.12,.02]
Openness	−.06 (.04)	[−.11,.04]

Note. N = 430. Standardized coefficients are displayed. Indirect effects do not have associated p‑values; significance was determined by whether the bias‑corrected 95% confidence interval excluded zero. ^a^SE = Standard error of the unstandardized coefficient (b). ^b^Bias-corrected 95% confidence interval based on 5,000 bootstrap samples. ^c^LL-UL = Lower limit-upper limit. ^d^PSMU = Problematic social media use. ^e^T1 = Time 1. ^f^T2 = Time 2. ^g^ab‑paths represent the indirect effects obtained through each mediator. ^h^The c‑path corresponds to the total effect of PSMU on suicidal ideation. ^i^The c′‑path reflects the direct effect after accounting for the mediators. * *p* <.05. ** *p* <.01. *** p <.001

## Discussion

The growing interest in PSMU as a predictor of suicidal ideation in adolescence [3, 29] highlights the need to explore the underlying mechanisms and associated risk factors, and this prospective study makes a significant contribution to the existing literature by identifying maladaptive CERS—specifically self-blame— as a mediating mechanism in the relationship between PSMU and suicidal ideation.

In relation to the first hypothesis, this study found a positive association between PSMU and suicidal ideation nine months later, in line with prior research [3, 20], a finding that aligns with existing evidence on the detrimental effects of excessive use of social media on mental health [16] and, more specifically, on the risk of suicide [29]. Furthermore, PSMU was positively associated with maladaptive CERS, supporting previous findings that link emotion regulation difficulties to externalizing problems and problematic behaviors, including excessive use of social media [64–66].

In line with previous research, maladaptive CERS were prospectively correlated with suicidal ideation, with self-blame showing the strongest correlation, followed by catastrophizing and rumination. The impact of self-blame and rumination in suicide-related outcomes aligns with prior studies [67], as well as with findings on depression, a well-established risk factor for suicidality [33, 68]. Specifically, individuals experiencing depression tend to attribute negative events to internal, stable, and global factors, reinforcing self-blame even in the absence of objective wrongdoing [68]. Similarly, catastrophizing has been linked to anxiety, depression [69], and nonsuicidal self-injury [70], further supporting its contribution to an elevated risk of suicidality. An unexpected result, meanwhile, was the lack of a prospective association between other-blame and suicidal ideation. Although empirical studies in adolescents are scarce, this finding reinforces prior research indicating that cognitive control deficits influence through self-blame, acceptance, rumination, and catastrophizing, but not other-blame [48]. During adolescence, ongoing identity development and increased sensitivity to social evaluation shape self-evaluative processes, marking this period as a sensitive phase for self-concept development [71]. Within this developmental context, internally oriented cognitive processes, including self-blame, self-criticism, and rumination, have been associated with internalizing psychopathology in adolescence, with evidence linking self-blame to internalizing symptoms [72], self-criticism to depressive and anxiety-related outcomes [73], and rumination to the maintenance of negative self-focused processing and internalizing symptomatology [74]. In contrast, externally oriented attributional tendencies, such as hostile attribution or externalization of blame, have been more strongly associated with externalizing outcomes, including aggression and disruptive behavior [75, 76]. Accordingly, the absence of a significant association of other-blame in the present study may reflect the developmental specificity of internally oriented cognitive mechanisms during adolescence, which appear to be more associated with internalizing symptomatology than externally directed attributional styles at this stage of development.

Additionally, in the context of childhood sexual abuse, self-blame has been associated with poorer long-term psychological adjustment (e.g., low self-esteem, relationship anxiety), whereas attributing blame to the perpetrator was not significantly related to later adjustment [77]. Supporting this, Barker-Collo (2001) found that internal attributions of blame during childhood significantly predicted suicide attempts in adulthood [67]. Based on this evidence, it is plausible that self-focused cognitive distortions, such as self-blame and rumination, along with catastrophizing, play a critical role in the development and maintenance of suicidal thoughts in adolescents over time; however, further research is needed to explore these prospective relationships between CERS and suicidal ideation.

Our results partially supported the second hypothesis, with self-blame emerging as the only significant mediator between PSMU and suicidal ideation. This finding is consistent with theories suggesting that internalized blame exacerbates the effects of negative emotions, heightening vulnerability to psychological issues such as suicidal behavior [48] and ideation [49]. In the context of social media, it is possible that PSMU may intensify self-critical cognitive processes, such as constant social comparison and excessive concern about online interactions, which in turn contribute to self-blame [50]. The mediating role of self-blame can be contextualized within broader theoretical frameworks and empirical findings. According to social comparison theory [45], individuals evaluate their worth by comparing themselves to others. On social media, adolescents are frequently exposed to idealized portrayals of perfect lives of peers and influencers [46], and these curated depictions often emphasize achievements, positive emotions, and happiness, creating a stark contrast with adolescents’ internal struggles, such as the reinforcement of feelings of being different that can relate to suicidal thoughts. This dissonance can lead to feelings of inferiority, dissatisfaction, and heightened self-blame [46, 78], and the latter may further exacerbate this cycle by intensifying negative self-perceptions. Adolescents may attribute their inability to meet these idealized standards to personal failings, reinforcing negative emotions such as guilt, shame, and self-disgust, which, in turn, are positively related to suicidality [79]. This is in accordance with the interpersonal theory of suicide [6], in which self-blame and a sense of being neglected by others are elements that might contribute to thwarted belongingness and perceived burdensomeness (which comprises a degree of self-hatred, as evidenced by the existence of self-blame) [48, 49]. In sum, PSMU seems to act as a catalyst for self-blame by amplifying exposure to unrealistic standards and triggering maladaptive cognitive processes that lead to negative emotional responses, prompting a desire to avoid both self-awareness and the emotional distress it entails in triggering suicidal thoughts. This highlights the importance of addressing self-blame as a modifiable mechanism in prevention and interventions targeting the mental health effects of PSMU, especially related to suicidal thoughts. Nonetheless, although the present longitudinal findings support the proposed pathway from PSMU to self-blame and, in turn, to suicidal ideation, alternative directional processes should also be considered. In line with the self-medication hypothesis [80], adolescents with elevated self-blame or suicidal ideation may engage in PSMU as a compensatory emotion-regulation. From this perspective, PSMU may operate not only as a risk factor but also as a maladaptive coping response to pre-existing psychological vulnerability, underscoring the need for future studies to further clarify directionality.

The findings not only enhance our understanding of the effects of PSMU on suicidal ideation in a sample of nonclinical adolescents but also provide evidence of a mediating mechanism that can be considered a significant adjunct to existing prevention and intervention strategies [81, 82]. From an educational perspective, programs should prioritize fostering digital literacy and promoting conscientious use of social media in adolescents [46]. These initiatives can empower adolescents to critically evaluate the curated and often idealized content they encounter online, thereby reducing the detrimental impact of harmful social comparisons [78]. In this context, school-based interventions may benefit from incorporating brief, structured activities aimed at identifying maladaptive cognitions, such as self-blaming thoughts related to online peer interactions, which can subsequently be addressed through cognitive restructuring strategies. These strategies, including evidence checking, generation of alternative attributions, and decatastrophizing, have been successfully embedded in short, CBT-informed educational programs targeting cyberbullying or social media use in school settings [83, 84]. In parallel, emerging evidence suggests that promoting more adaptive patterns of social media engagement, such as mindful use, may help adolescents develop a healthier relationship with social media [85]. Taken together, the integration of digital literacy components with cognitive restructuring techniques may enhance adolescents’ capacity to reinterpret self-blaming narratives arising from online interactions and reduce their potential impact on psychosocial adjustment. Moreover, integrating modules that cultivate self-forgiveness and self-compassion within emotional education programs tailored to the digital context could further mitigate the adverse psychological effects of social media use and contribute to improved mental health outcomes. From a clinical perspective, although our results need to be replicated in clinical samples, prioritizing the recognition of self-blaming narratives and fostering a more compassionate and forgiving view of themselves seem to be keys when working with adolescents.

### Limitations and future considerations

The present study is not without limitations. First, although suicidal ideation is a critical construct, it is not the sole predictor of suicide, and future studies should explore the relationship between PSMU, CERS, and broader suicidality factors, such as suicide attempts or behaviors, as well as other internalizing problems linked to PSMU, such as nonsuicidal self-injury. Additionally, it is crucial to include clinical or high-risk community samples of adolescents to examine whether these findings extend to populations at greater risk of suicide ideation. Second, the study did not account for other relevant affective disorders, such as depression or low self-esteem, or stressful relational challenges (e.g., parental divorce, romantic break-up, (cyber)bullying, recent loss of a loved one, …) [86, 87], which may act as confounding variables and/or can act as relevant mediators between problematic social media use and self-harm behaviors (e.g., affective disorders [88]),. Controlling and integrating these factors into future research would provide a more comprehensive understanding of the consequences of PSMU. Third, this study relied solely on self-reported measures, which may be subject to biases such as social desirability or recall bias. Moreover, although the internal consistency of some CERQ-SA subscales was at the lower bound of acceptability, such values are commonly reported for short subscales and exploratory research and should therefore be considered when interpreting the magnitude of the observed associations. Incorporating repeated prospective assessments and within-person designs would enhance the reliability of the findings and provide deeper insights into the coping patterns involving self-blame and suicidal ideation. Fourth, although our results showed small-to-medium effect sizes between PSMU and suicidality, these findings are consistent with prior research [19, 29] and suggest the possibility of person-specific effects [89]. Fifth, some evidence suggests that adolescents at risk of suicidality may use social media and smartphones excessively as a coping mechanism, rather than social media being a direct cause of suicidality [29], as well as maladaptive emotion regulation leading to PSMU [28, 65]. Future research should explore these reverse or bidirectional relationships to clarify the nature of these associations. Finally, the reduction in sample size from T1 to T2 represents a limitation that may affect the generalizability of the longitudinal findings. However, the pattern of attrition largely reflects structural features of the school context, rather than systematic dropout related to the study variables.

## Conclusions

This study identified self-blame as a critical mediator in the relationship between PSMU and suicidal ideation over time. Given that CERS are modifiable, interventions targeting self-blame hold promise for mitigating the adverse effects of PSMU on suicidal ideation. Further longitudinal studies with diverse samples are needed to validate these findings and explore bidirectional or recursive relationships between PSMU, self-blame, and suicidal thoughts.

## Data Availability

Anonymized data collected is available as open data via the University of Málaga online data repository https://riuma.uma.es/xmlui/handle/10630/39918.
